# Social Connectedness Risk Among Middle-Aged and Older Black Men With Chronic Conditions

**DOI:** 10.1093/geroni/igab046.2068

**Published:** 2021-12-17

**Authors:** Matthew Smith, Ledric Sherman, Kirby Goidel, Thomas Cudjoe

**Affiliations:** 1 Texas A&M School of Public Health, College Station, Texas, United States; 2 Texas A&M University, College Station, Texas, United States; 3 Johns Hopkins University School of Medicine, Baltimore, Maryland, United States

## Abstract

Social connectedness is a multi-factorial concept encompassing structural, functional, and quality aspects. For those with chronic conditions, threats to social connectedness can exacerbate illness symptomology and impose barriers to disease self-management. This study identified factors associated social connectedness risk among Black men ages 40+ years with one or more chronic conditions. Data from 2019 were analyzed from a national sample of 1,200 Black males collected with an internet-delivered questionnaire. Three logistic regression models were fitted to assess factors associated with not having enough people to call for help (structural), feeling isolated from others (functional), and not being content with friendships/relationships (quality). All regression models were adjusted for age, education, marital status, number of chronic conditions, self-reported barriers to disease self-management, and having cut-down or skipped social activities because of health problems. On average, participants were age 56.7 (±9.7) years and self-reported 4.0 (±2.9) chronic conditions. Approximately 23% were ages 65+ years, 45.4% cut-down or skipped social activities because of health problems, 25.2% did not have enough people to call for help, 26.0% felt isolated from others, and 23.8% were not content with friendships/relationships. Across the three regression models, men who were middle-aged (P<0.05), never married (P<0.01), cut-down or skipped social activities because of health problems (P<0.001), and reported more barriers to disease self-management (P<0.001) were significantly more likely to report social connectedness risk. Findings suggest that efforts to improve the self-management of illness symptomology may mitigate threats to structural, functional, and quality aspects of social connectedness among this male population.

